# Effect of Qufengtongluo Decoction on PI3K/Akt Signaling Pathway in the Kidney of Type 2 Diabetes Mellitus Rat (GK Rat) with Diabetic Nephropathy

**DOI:** 10.1155/2018/8421979

**Published:** 2018-01-14

**Authors:** Wei-Jun Huang, Qiang Fu, Yong-Hua Xiao, Qing Gong, Wen-Jing Wu, Zi-Long Shen, Hua Zhang, Xu Jia, Xue-Min Huang, Ya-Xin Zhang, Jin-Xi Zhao, Shi-Dong Wang, Mian Jia, Yu-Ting Zhang

**Affiliations:** ^1^Section II of Endocrinology & Nephropathy, Department of Dongzhimen Hospital Affiliated to Beijing University of Chinese Medicine, Beijing University of Chinese Medicine, Beijing, China; ^2^Nephropathy Department, Hubei Provincial Hospital of TCM, Hubei University of Chinese Medicine, Hebei, China; ^3^Nephropathy Department, Beijing Hospital of Traditional Chinese Medicine Affiliated to Capital Medical University, Capital Medical University, Beijing, China; ^4^Scientific Research Experiment Center, Beijing University of Chinese Medicine, Beijing, China; ^5^Department of Integrative Traditional Chinese Medicine and Western Medicine, Peking University First Hospital, Beijing, China; ^6^Department of Rehabilitation, Beijing Sport University, Beijing, China

## Abstract

Qufengtongluo (QFTL) decoction is an effective treatment for diabetic nephropathy (DN). However, the underlying molecular mechanism is still unclear. In this study, we try to investigate whether QFTL decoction acts via inhibiting PI3K/Akt signaling pathway. Twenty-four GK rats were randomly divided into 3 groups: blank group, sham-operated group, and QFTL group. After model establishment, rats in QFTL group were given QFTL decoction by gavage, while the rest were given pure water. During the 8-week intervention, 24 hr urinal protein was measured every 2-3 weeks. After intervention, kidneys were removed for pathological smear, quantitative real-time PCR, and western blotting to detect expression levels of p-PI3K, p-Akt, PTEN, TGF-*β*, PI3K mRNA, Akt mRNA, PTEN mRNA, and TGF-*β* mRNA. QFTL group showed a slighter degree of renal fibrosis in Masson and PASM staining and a greater reduction of 24 hr urinal protein than blank group. Compared to blank group, expression levels of p-PI3K, p-Akt, PI3K mRNA, and Akt mRNA were lower in QFTL group, while expression levels of PTEN and PTEN mRNA were higher. Besides, TGF-*β* was downregulated by QFTL decoction. In conclusion, this study suggests that QFTL decoction might inhibit PI3K/Akt signaling pathway via activating PTEN and inhibiting TGF-*β*.

## 1. Introduction

Diabetic nephropathy (DN) is one of the most significant diabetic microvascular complications of diabetes and currently the main cause of end-stage renal disease (ESRD) [1]. Beside allopathic therapeutic agents, traditional Chinese medicine (TCM) also offers effective methods in treating DN. In clinical practice, we find that many wind-medicines, which are used to dispel external wind pathogen, reduce the urinal protein of DN patients [2]. Among the wind-medicines, effect in decreasing urinal protein of* Tripterygium* has been proven [3]. Besides* Tripterygium*,* Niubangzi (Fructus Arctii)* [4],* Chuan Shan Long (Dioscoreae Nipponicae Rhizoma)*,* Can Sha (Silkworm Sand)* [5], and* sinomenine* are also used to treat diabetic kidney disease and these medicines constitute the ingredients of Qufengtongluo (QFTL) decoction. In clinical practice, we have observed that the Qufengtongluo (QFTL) decoction, which dispels wind and frees collateral vessels, has the effect of decreasing urinal protein. But the underlying molecular mechanism is still unclear.

PI3K/Akt is a common signaling pathway which promotes cell proliferation and inhibits apoptosis [6]. In recent years, it has been shown that PI3K/Akt signaling pathway plays an important role in the pathogenesis of DN, garnering much attention [7, 8]. Many drugs can treat DN by regulating PI3K/Akt signaling pathway [9–11]. In this study, we try to investigate whether QFTL decoction acts through PI3K/Akt signaling pathway.

## 2. Materials and Methods

### 2.1. Animals

Twenty-four male pathogen-free GK rats were purchased from Changzhou Cavens Laboratory Animal Co. Ltd. (age between 11 and 17 months, initial weight about 300 g, qualified number SCXK (SHU) 2011-0003). All animals were housed under standard conditions (constant ambient temperature of 22°C and humidity of 60% in a 12-h light/dark cycle) in the animal house of Beijing University of Chinese Medicine, with free access to water. The study followed the national guidelines for laboratory animal welfare and was approved by the Animal Ethics Committee of Beijing University of Chinese Medicine (number BUCM-4-2015071701-3001). Furthermore, in order to minimize animal suffering, the animals were sacrificed under anesthesia after the experiment.

### 2.2. Drugs and Reagents

QFTL decoction, composed of* Niubangzi (Fructus Arctii)*,* Chuan Shan Long (Dioscoreae Nipponicae Rhizoma)*,* Can Sha (Silkworm Sand)*, and* sinomenine*, was provided by Beijing Tcmages Pharmaceutical Co. Ltd. High-fat animal feeds, containing 10% lard, 2% cholesterol, 0.25% cholate, 5% saccharose, and 82.75% basal feed, were provided by Beijing KEAO XIELI Feed Co. Ltd. Pentobarbital sodium (Sigma-P3761, CAS 57-33-0) was bought from Beijing Think-Far Technology Co. Ltd. Penicillin g sodium for injection (lot number: F4092105) was provided by North China Pharmaceutical Co. Ltd. Phospho-PI3K p85 (Tyr458)/p55 (Tyr199) Antibody (Art. number 4228S) and Phospho-Akt (Ser473) (D9E) XP Rabbit mAb (Art. number 4060S) were purchased by Cell Signaling Technology. Anti-PTEN antibody (lotGR174512-1) and anti-TGF-*β* (lotGR134709-5) were provided by Abcam. Dako REALTM EnVision TM Detection System, Peroxidase/DAB+, and Rabbit/Mouse were offered by Danish Dako Company. Nuclear-Cytosol Extraction Kit, 5x SDS-PAGE loading buffer, 1.5 M Tris·HCl buffer, 1.0 M Tris·HCl buffer, 30% acrylamide, AP, TEMED, TBST, and 10x electrophoretic buffer were all supplied by Beijing Applygen Technologies Inc. BCA protein assay kit, RIPA Lysis Buffer, and PMSF were afforded by Beyotime Institute of Biotechnology. Multicolor protein marker, ECL Western Blotting Substrate, and TRIzol reagent were provided by Thermo Fisher Scientific. RT reagent kit and PCR primer were furnished by Dalian TaKaRa Bio Inc.

### 2.3. Equipment and Instruments

The following equipment and instruments were used: TB-718 Tissue embedding console system (Sakura Finetek Co. Ltd., Japan), Microtome RM2235 (Leica Microsystems GmbH, Germany), Leica HI1220 Slide drier (Leica Microsystems GmbH, Germany), Leica ST5020 Vacuum tissue processor (Leica Biosystems GmbH, Germany), Olympus bx51 microscope (Olympus corporation, Japan), Synergy 4 microplate reader (BioTek Instruments Inc., USA), IEC refrigerated centrifuge (Thermo Fisher Scientific, USA), AR2130 Electronic balance (OHAUS corporation, USA), Mini Trans-Blot Transfer (Bio-Rad Laboratories Inc. USA), Mini-PROTEAN3 cell (Bio-Rad Laboratories Inc. USA), DYY-10 Electrophoresis apparatus (Beijing LIUYI biotechnology Co., Ltd., China), TS-1 Decoloring shaker (Haimen city QiLin medical Instrument Factory, China), Image LabTM XRS + Gel image analysis and management system (Bio-Rad Laboratories Inc., USA), Real-time PCR cycler (Applied Biosystems Inc.), and Accu-Chek Performa blood glucometer (Hoffmann-La Roche Ltd., Switzerland).

### 2.4. Preparation of QFTL Decoction

The traditional Chinese medicine granules were dissolved in pure water. 1 mL of the liquid preparation of QFTL was given to the rat by gavage per 100 g body weight and the drug dose used for rats was 10 times the human dose (according to “pharmacological experimental methodology” edited by Professor Xu Shuyun), calculated according to the body weight. The concentration of QFTL decoction was 3.9 mg/ml (Niubangzi 0.25 mg/ml, Chuan Shan Long 0.5 mg/ml, Can Sha 0.15 mg/ml, and* sinomenine *3 mg/ml).

### 2.5. Establishing Models of DN

The DN model was established by feeding rats with a high-fat diet followed by unilateral nephrectomy. Firstly, rats were acclimatized for a period of one week prior to conducting experiments. Then rats were fed with high-fat diet for eight weeks. Following a 12 hr fasting period, oral glucose tolerance test (OGTT) (2 g/kg) was performed the next morning. Blood specimens were collected from the end of tail region and glucose content was measured using Accu-Chek Performa blood glucometer. Then, 24 rats were equally randomized to three groups (blank group, sham-operated group, and QFTL group) by stratified randomization, based on the 2-hour postprandial blood glucose (2hPG = 11.1 mmol/l) level. After the stratified randomization, blank group and QFTL group received a unilateral nephrectomy, while sham-operated group received a sham operation (cut the corresponding skin and muscle on the back then sutured). The operation was conducted under anesthesia by intraperitoneal injection of 3% pentobarbital sodium (1.5 mL/kg). After operation, rats were allowed to recover in a warm and clean cage and Penicillin (400000 units for each rat) was injected for three consecutive days.

### 2.6. Treatment Administration

Treatment began one week after recovery. QFTL group was given QFTL decoction by gavage, while blank group and sham-operated group were given pure water (1 mL per 100 g body weight). The drug intervention lasted for 8 weeks. During the intervention period, all rats were given a high-fat diet.

### 2.7. Test of 24 hr Urinal Protein

24 hr urine samples from rats were collected with metabolic cages before and after the treatment (the zeroth week, the third week, the fifth week, and the eighth week). After urine collection, the urine volume was measured using a volumetric cylinder, and urinal protein concentration was tested using coomassie brilliant blue (CBB). Values of the total urine volume and protein concentration were used to calculate the 24 hr urinal protein.

### 2.8. Kidney Removal

In order to examine the renal histopathological changes and explore the underlying molecular biological mechanism, the remaining kidney was removed after intervention. The removed kidney was cut sagittally and half the kidney was fixed in 10% paraformaldehyde for HE staining, Masson staining, and PASM staining, while the other half was kept in a cryotube for western blotting, immunohistochemistry staining, and RT-PCR. The kidney removal and blood collection were performed under 3% pentobarbital sodium anesthesia.

### 2.9. Western Blotting to Detect p-PI3K, p-Akt, and PTEN in the Kidney Tissue

First, about 50 mg kidney tissue was ground into powder in the mortar. Then, 1 mL RIPA was added and the tissue lysis lasted for 30 min in the ice. After tissue lysis, liquid supernatant was transferred into the EP tube to centrifuge under 4°C. Then, the liquid supernatant was subpackaged into new EP tubes and all EP tubes were stored in the −20°C refrigerator. BCA (bicinchoninic acid) method was used to detect protein concentration, following the introduction of BCA kit. The loading volume was calculated according to the protein concentration. After the preparation of SDS-PAGE gel, 20 ug of protein samples were loaded into the wells. After loading, the samples were run under constant voltage of 60 v for 20 min and then 110–120 v until the leading edge of dye was near the bottom of gel. After electrophoresis, membrane transfer was conducted under the constant current of 80–120 mA for 100 min. Then the membrane was blocked with 5% BSA for 30 min. After blocking, the primary antibody was added and overnighted in the 4°C refrigerator. Then the membrane was rinsed with TBST for 5 times and 9 min each time. After blocking again with 5% BSA, secondary antibody (1 : 20000) was added and incubated for 1.5 hr. The chemiluminescence coloration began after the secondary antibody incubation was completed. The protein bands were analyzed by the Image Lab software.

### 2.10. Quantitative Real-Time PCR to Detect the PI3K mRNA, Akt mRNA, and PTEN mRNA

(a) Preparation of RNA: the RNA was extracted from 50 mg kidney tissue and kept in −70°C refrigerator for PCR. RNA concentration and OD260/OD280 were measured using a spectrophotometer. (b) Reverse transcription: after preparing the reverse transcription reaction mixture (RNase Free ddH_2_O 3.0 *μ*L, 5x Primer Script Buffer 4.0 *μ*L, 50 *μ*M Oligo dT Primer 1.0 *μ*L, 100 *μ*M Random 6 mers 1.0 *μ*L, Primer Script RT Enzyme Mix I 1.0 uL, and 200 ng/*μ*L total RNA 10.0 uL), the reverse transcription reaction was carried out in PCR machine (reverse transcription reacted for 15 min under 37°C and reverse transcriptase enzyme inactivated for 5 s under 85°C). Then, the cDNA was refrigerated at −20°C. (c) Quantitative real-time PCR: primers (provided by Takara) contained PI3K upstream primer (5′AGATGCTTTCAAACGCTAT3′), PI3K downstream primer (5′GCTGTCGCTCACTCCA3′), Akt upstream primer (5′TCTATGGCGCTGAGATTGTG3′), Akt downstream primer (5′ CTTAATGTGCCCGTCCTTGT3′), PTEN upstream primer (5′TTGAAGACCATAACCCACCACA3′), PTEN downstream primer (5′ATATCATTACACCAGTTCGTCCCT3′), TGF-*β* upstream primer (5′GGGACTATCCACCTGCAAGA3′), downstream primer (5′CCTCCTTGGCGTAGTAGTCG3′), *β*-actin upstream primer (5′ACGTTGACATCCGTAAAGACC3′), and *β*-actin downstream primer (5′GCCACCAATCCACACAGAGT3′). The reaction system had been prepared before PCR. It contained 10 *μ*M forward primers 0.5 *μ*l, 2x UltraSYBR Mixture 10.0 *μ*l, ddH_2_O 7.0 *μ*l, and cDNA 2.0 *μ*l. The real-time PCR reaction was performed on the ABI 7500 Fast. The genes obtained from the kidney samples each had three wells, including a negative control well. Initial denaturation lasted for 5 min under 95°C. Then gene amplification lasted for 15 s under 95°C and 20 s under 60°C. It was repeated for 40 cycles and the fluorescence amplification curve and melting curve were collected after each cycle. The 2^−ΔΔCT^ method was used to analyze the data.

### 2.11. Immunohistochemistry Method to Detect the p-PI3K, p-Akt, and PTEN in the Kidney Tissue

Kidney paraffin sections were dewaxed by xylene and alcohol of different concentrations. The high-pressure antigen retrieval method was used for antigen retrieval. 3% H_2_O_2_ was used to inactivate endogenous peroxidase. After endogenous peroxidase ablation, primary and secondary antibodies were added to the section successively. After coloration with 3,3-diaminobenzidine (DAB), the sample was stained with hematoxylin. Samples were mounted with neutral gums after dehydration. Images were analyzed using Image-Pro Plus 5.1 system.

### 2.12. Statistical Analysis

All statistical analyses were performed using SPSS Statistics 20 software (IBM Corporation, Armonk, NY, USA). Data were expressed as mean ± standard deviation ($\overset{-}{x}\pm SD$). Normally distributed data were analyzed using one-way ANOVA and nonnormal distributions were analyzed by nonparametric tests. Statistical differences were considered as significant if the *P* value was less than 0.05.

## 3. Results

### 3.1. The Survival of Rat

Rats of sham-operated group had better vitality, shinier coat, and more obvious weight-gain than rats of blank and QFTL group. All rats had completed the study except for one rat in sham-operated group, which died after enduring massive hemorrhage from the one-sided nephrectomy.

#### 3.1.1. HE Staining

**Figure 1:**
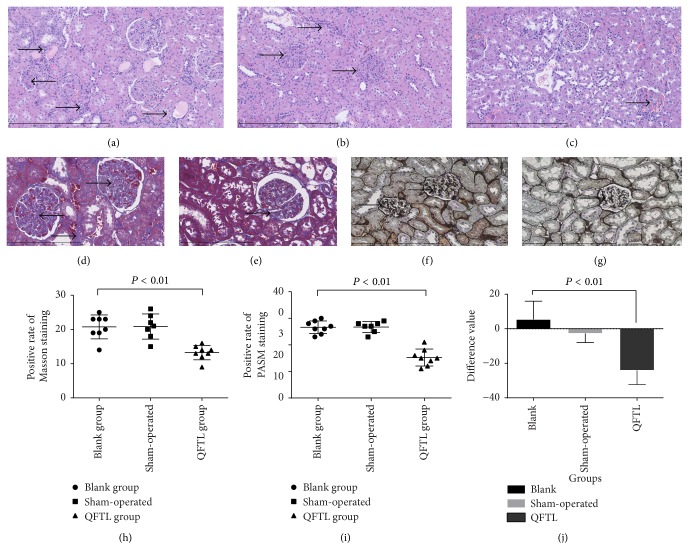
* HE staining, Masson staining, PASM staining, and 24 hr urinal protein*. Histological changes of blank group (*n* = 8), sham-operated group (*n* = 7), and QFTL group (*n* = 8) were observed by HE staining, Masson staining, PASM staining. When the Masson and PASM staining were quantified, three fields of each section were randomly chosen. Mean positive rate of these three fields was calculated to stand for the positive rate of whole sample. (a) Glomerular pathological changes of blank group (glomerular hypertrophy, angiotelectasis, mesangial cell proliferation, mesangial matrix expansion, glomerular sclerosis, narrowed Bowman's capsule, vacuolar degeneration of tubular epithelial cells, tube casts, inflammatory cell infiltration, and fibrous tissue proliferation). (b) Glomerular pathological changes of sham-operated group (glomerular hypertrophy, angiotelectasis, mesangial cell proliferation, mesangial matrix expansion, Bowman's capsule narrowing, vacuolar degeneration of tubular epithelial cells, inflammatory cell infiltration, and fibrous tissue proliferation). (c) Glomerular pathological changes of QFTL group (glomerular hypertrophy, angiotelectasis, mesangial cell proliferation, mesangial matrix expansion, Bowman's capsule narrowing, multifocal renal tubular epithelial cell vacuolar degeneration, and derangement of kidney tubules). (d) Masson staining of blank group. (e) Masson staining of QFTL group. (f) PASM staining of blank group. (g) PASM staining of QFTL group. (h) The positive rate of Masson staining: the mean positive rate of blank group was notably higher than that of QFTL group. (i) The positive rate of PASM staining: the mean positive rate of blank group was notably higher than that of QFTL group. (j) The difference value of 24 hr urinal protein.

HE staining was used to assess the glomerular volume, glomerular capillary loops, mesangial area, renal tubular epithelial cells, and renal interstitium. The renal structure was observed at a magnification of 100x and 200x. The major glomerular pathological changes seen in sham-operated group were glomerular hypertrophy, angiotelectasis, mesangial cell proliferation, mesangial matrix expansion, mild glomerular sclerosis, Bowman's capsule narrowing, vacuolar degeneration of tubular epithelial cells, mild infiltration of inflammatory cell in the renal interstitium, and a small amount of fibrous tissue proliferation (Figure 1(b)). Compared to sham-operated group, blank group had more serious pathological changes. More glomerular sclerosis, Bowman's capsule narrowing, and tube casts could be observed in the renal biopsy of rats in blank group (Figure 1(a)). However, QFTL group had comparatively minimal pathological changes, with no glomerular sclerosis and no tube casts present. The pathological changes were limited to glomerular hypertrophy, minimal mesangial cell proliferation and mesangial matrix expansion, multifocal renal tubular epithelial cell degeneration, and mild derangement of kidney tubules (Figure 1(c)).

#### 3.1.2. Masson Staining and PASM Staining

Masson and PASM staining were used to examine the thickness of glomerular basement membrane and mesangial matrix. The rat kidney section was observed under a microscope of 200x magnification. Results showed that the glomerular basement membrane and mesangial matrix of the blank and sham-operated groups had severe hyperplasia, while the glomerular basement membrane and mesangial matrix of QFTL group had slight hyperplasia (Figures 1(d)–1(g)). The positive rate of Masson staining conformed to the normal distribution except that of sham-operated group (*P* > 0.05). So, one-way ANOVA was used to analyze the mean difference between three groups. Statistical analysis showed that the mean positive rate of blank group was notably higher than that of QFTL group (*P* < 0.01, Figure 1(h)).

The positive rate of PASM staining conformed to normal distribution in all groups and one-way ANOVA was used to analyze the intergroup difference. As a result of Masson staining, the mean positive rate of blank group was notably higher than that of QFTL group (*P* < 0.01, Figure 1(i)).

### 3.2. 24 hr Urinal Protein

The 24 hr urinal protein was tested in the zeroth week, the third week, the fifth week, and the eighth week. The difference between 24 hr urinal protein levels in zeroth week and eighth week was analyzed. The data of each group followed a normal distribution (*P* > 0.05), and one-way ANOVA was used to analyze the intergroup difference (Figure 1(j)). There was no significant difference between sham-operated group and blank group (*P* > 0.05). However, there was a significant reduction in 24 hr urinal protein levels of rats in QFTL group (*P* < 0.01).

The results mentioned in Figure 1 suggested that QFTL decoction was effective in treating DN. But what was the underlying molecular mechanism? To uncover the molecular mechanism, we detected the expression level of p-Akt, p-PI3K, and PTEN. The mRNA of these three biological factors was also tested by qPCR.

### 3.3. Immunohistochemistry Method to Detect the Expression of p-PI3K, p-Akt, PTEN, and TGF-*β* in the Kidney Tissue

**Figure 2:**
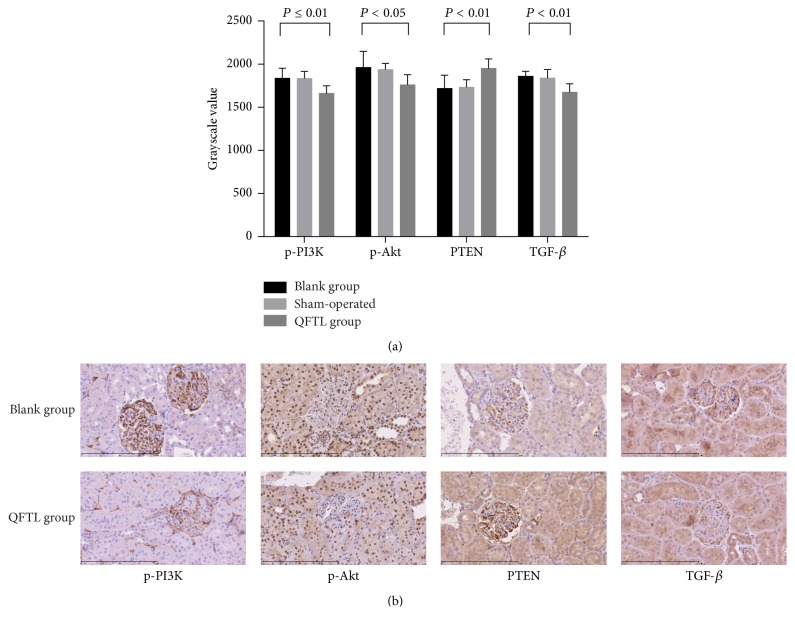
Immunohistochemistry results. Three fields were captured from one section and average grayscale value was calculated, which stood for the grayscale value of the sample. The p-PI3K was mainly expressed in the mesangial cell. PTEN and TGF-*β* were mainly in the cytoplasm of mesangial and renal tubular epithelial cells, while p-Akt localized in the nucleus of mesangial and renal tubular epithelial cells. (a) The grayscale value of p-PI3K, p-Akt, PTEN, and TGF-*β*. The grayscale vale of *y*-axis stands for averages of blank group (*n* = 8), sham-operated group (*n* = 7), and QFTL group (*n* = 8). (b) Images of immunohistochemistry.

Results showed that the brown positive staining was mainly seen in the mesangial and renal tubular epithelial cells. Both the cytoplasm and nucleus could be positively stained. The p-PI3K was mainly expressed in the mesangial cell. PTEN and TGF-*β* were mainly in the cytoplasm of mesangial and renal tubular epithelial cells, while p-Akt localized in the nucleus of mesangial and renal tubular epithelial cells (Figure 2(b)). The grayscale value was detected to evaluate expression levels of p-PI3K, p-Akt, PTEN, and TGF-*β* (Figure 2(a)). Because the data of p-PI3K and p-Akt in blank group did not conform to the normal distribution (*P* < 0.05), a nonparametric test was used to analyze the mean difference. Results showed that expression levels of p-PI3K (*P* ≤ 0.01), p-Akt (*P* < 0.05), and TGF-*β* (*P* < 0.01) were significantly lower in QFTL group than those in blank group. However, expression level of PTEN in QFTL group was much higher than that of blank group (*P* < 0.01).

### 3.4. Western Blotting to Detect p-PI3K, p-Akt, PTEN, and TGF-*β* in the Kidney Tissue

**Figure 3:**
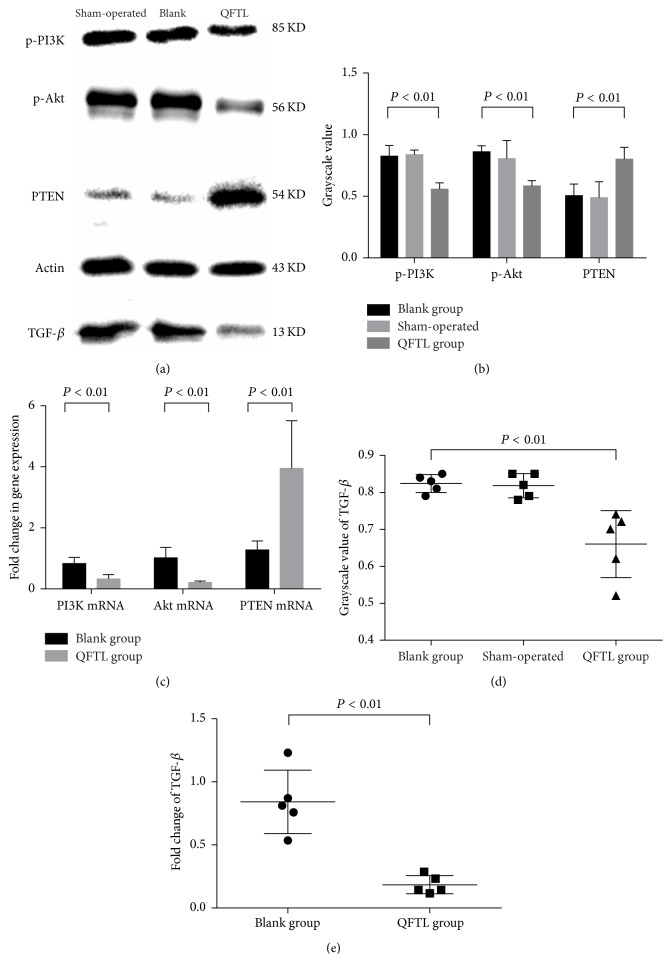
Results of quantitative real-time PCR and western blotting. Five samples were randomly chosen from each group to do the western blotting and real-time PCR. (a) Representative protein bands of p-PI3K, p-Akt, PTEN, and TGF-*β*. (b) Grayscale values (*n* = 5) of p-PI3K, p-Akt, and PTEN. (c) Fold changes (*n* = 5) of PI3K mRNA, Akt mRNA, and PTEN mRNA expression of blank group and QFTL group. (d) Grayscale values (*n* = 5) of TGF-*β*. (e) Fold changes (*n* = 5) of TGF-*β* mRNA expression of blank group and QFTL group.

Western blotting was used to detect the expression levels of p-PI3K, p-Akt, and PTEN proteins. Protein bands with a relative molecular weight of 85 kDa (p-PI3K), 56 kDa (p-Akt), 54 kDa (PTEN), and 13KD (TGF-*β*) could be seen (Figure 3(a)). One-way ANOVA was used to analyze the grayscale value (Figures 3(b) and 3(d)). There was no significant difference between the sham-operated and blank groups in expression levels of p-PI3K (*P* ≥ 0.05), p-Akt (*P* ≥ 0.05), PTEN (*P* ≥ 0.05), and TGF-*β* (*P* ≥ 0.05). However, there was a significant difference in the expression levels of p-PI3K (*P* ≤ 0.001), p-Akt (*P* ≤ 0.001), PTEN (*P* ≤ 0.01), and TGF-*β* (*P* ≤ 0.01) when comparing QFTL group with blank group.

### 3.5. Quantitative Real-Time PCR to Detect the Expression of PI3K mRNA, Akt mRNA, PTEN mRNA, and TGF-*β*

Assessment of mRNA was performed by quantitative real-time PCR, as previously described. The data of sham-operated group was used as the calibrator for data conversion. As seen in Figures 3(c) and 3(e), expression levels of PI3K mRNA (*P* ≤ 0.01), Akt mRNA (*P* ≤ 0.001), and TGF-*β* mRNA (*P* ≤ 0.01) in QFTL group were much lower than those of blank group. However, the PTEN gene expression was more in QFTL group than blank group (*P* ≤ 0.01).

## 4. Discussion

DN is presently a great threat to health and TCM is a good treatment for this disease. In clinical practice, QFTL decoction has been observed to have a good curative effect for DN and we speculated that the effectiveness may pertain to anti-inflammation and anti-immune effects [12]. According to the previous clinical studies, some wind-medicines are effective in treating diabetic kidney disease. The results of one study from Guanzhou [2] showed that TCM decoction with wind-medicines could significantly decrease the urinary albumin excretion rate and urine albumin-to-creatinine ratio in III phase diabetic kidney disease patients (1984 Mogensen staging). From another clinical randomized controlled trial [13], results demonstrated that the* Sanhuang Yishen* granule, which contains wind-medicines, could affect better in decreasing 24 hr urinal protein than* irbesartan* in IV phase diabetic kidney disease patients. In this study, we observed that QFTL decoction significantly reduced the 24 hr urinal protein (Figure 1(j)) and more protein cast could be observed in HE sections (Figure 1(a)), which were in accordance with previous studies and our clinical observation, suggesting that QFTL decoction could effectively treat DN. Additionally, results of HE staining, Masson staining, and PASM staining showed that the degree of renal fibrous tissue hyperplasia in QFTL group was significantly lower than that of blank group (Figures 1(a)–1(i)), which suggested that QFTL decoction could not only reduce the urinal protein but also could reduce renal fibrosis.

P-PI3K, p-Akt, and PTEN are key players in PI3K/Akt signaling pathway. P-PI3K and p-Akt activate PI3K/Akt signaling pathway, while PTEN can inhibit PI3K/Akt signaling pathway [14]. PI3K/Akt signaling pathway closely contacts with the expansion of mesangial cell (MC) [8, 15, 16], podocyte apoptosis [17], and renal tubule injury [18, 19]. Moreover, several studies [8, 15, 20, 21] have shown that many inflammatory and immune factors, transforming growth factor-*β*1 (TGF-*β*1) [8, 15, 20] and tumor necrosis factor-a (TNF-a) [21], for example, contribute to DN formation through PI3K/Akt signaling pathway. In addition, many traditional Chinese medicines have been proved to act through PI3K/Akt signaling pathway. Zhou et al. [22] proved that Shen-Yuan-Dan Capsule (SYDC) could exert an antiatherosclerotic effect on ApoE^−/−^ mice fed with a high-fat diet. The action mechanism of SYDC was attributed to its ability to inhibit inflammatory reaction by regulating IRS-1/PI3K/Akt/NF-*κ*B signaling pathway. Hong et al. [23] certified that Jiangtang decoction ameliorates diabetic nephropathy through the regulation of PI3K/Akt-mediated NF-*κ*B pathways in KK-Ay mice. Wang et al. [24] also testified that Qiliqiangxin could attenuate anoxia-induced injuries in cardiac microvascular endothelial cells via NRG-1/ErbB signaling pathway which was most probably dependent on PI3K/Akt/mTOR pathway. In this study, we detected expression levels of p-PI3K, p-Akt and PTEN using immunohistochemistry (Figure 2) and western blotting (Figures 3(a)-3(b)). Results showed that expression levels of p-PI3K and p-Akt in QFTL group were much lower when compared to blank group while PTEN expression was much higher after treatment with QFTL decoction. The expression of PI3K mRNA, Akt mRNA, and PTEN mRNA was detected using quantitative real-time PCR. Results concurred with expression levels of p-PI3K, p-Akt, and PTEN (Figure 3(b)). Besides, TGF-*β* is an important upstream activator of PI3K/Akt signaling pathway [25, 26] and plays an important role in the formation of diabetic nephropathy [27–29]. The results of this study showed that QFTL decoction can also inhibit the level of TGF-*β* (Figures 2, 3(d), and 3(e)). All these results prompted that QFTL decoction might inhibit PI3K/Akt signaling pathway via activating PTEN and inhibiting TGF-*β*.

The DN model of this study was established by feeding rats with a high-fat diet followed by unilateral nephrectomy. Although some pathological manifestations (Figures 1(a)–1(c)) of DN could be seen under microscope, we did not observe the Kimmelstiel-Wilson Node, which can been found in the kidney tissue of level III DN [30]. It demonstrated that the condition of DN model was not serious enough. Thus, better DN model is expected in future studies. However, results of this study showed that QFTL decoction could reduce 24 hr urinal protein via inhibition of PI3K/Akt signaling pathway. But its specific regulation of downstream molecular of PI3K/Akt signaling pathway is still unclear.
